# Targeted inhibition of Polo‐like kinase 1 by a novel small‐molecule inhibitor induces mitotic catastrophe and apoptosis in human bladder cancer cells

**DOI:** 10.1111/jcmm.13018

**Published:** 2016-11-23

**Authors:** Zhe Zhang, Guojun Zhang, Chuize Kong

**Affiliations:** ^1^ Department of UrologyThe First Hospital of China Medical UniversityShenyang CityChina; ^2^ Department of HematologyShengjing Hospital of China Medical UniversityShenyang CityChina

**Keywords:** PLK1, RO3280, mitotic catastrophe, cell cycle, cell death

## Abstract

Bladder cancer is a common cancer with particularly high recurrence after transurethral resection. Despite improvements in neoadjuvant chemotherapy, the outcome of patients with advanced bladder cancer has changed very little. In this study, the anti‐tumour activities of a novel Polo‐like kinase 1 (PLK1) inhibitor (RO3280) was evaluated *in vitro* and *in vivo* in the bladder carcinoma cell lines 5637 and T24. MTT assays, colony‐formation assays, flow cytometry, cell morphological analysis and trypan blue exclusion assays were used to examine the proliferation, cell cycle distribution and apoptosis of bladder carcinoma cells with or without RO3280 treatment. Moreover, real‐time RT‐PCR and Western blotting were used to detect the expressions of genes that are related to these cellular processes. Our results showed that RO3280 inhibited cell growth and cell cycle progression, increased Wee1 expression and cell division cycle protein 2 phosphorylation. In addition, RO3280 induced mitotic catastrophe and apoptosis, increased cleaved PARP (poly ADP‐ribose polymerase) and caspase‐3, and decreased BubR1 expression. The *in vivo* assay revealed that RO3280 retarded bladder cancer xenograft growth in a nude mouse model. Although further laboratory and pre‐clinical investigations are needed to corroborate these data, our demonstration of bladder cancer growth inhibition and dissemination using a pharmacological inhibitor of PLK1 provides new opportunities for future therapeutic intervention.

## Introduction

Bladder cancer is the second most common malignancy of the genitourinary tract after prostate cancer. Although the early diagnosis and treatment has substantially improved, bladder cancer‐related mortality is still high. In China, bladder cancer is more prevalent than other urinary tract cancers and is one of the most common malignancies, accounting for 3.2% of all cancers. There are many risk factors involved in the formation of bladder tumours 1, 2, 3, 4, 5, including exposure to arylamines, smoking, low fluid intake, chronic urinary infection due to Schistosomal haematobium infection, low N‐acetyltransferase activity and the frequent use of hair dye. More than 90% of bladder cancers are transitional cell carcinomas (TCCs). Approximately 80% of all TCCs initially develop along the superficial papillary pathway; the remaining 20% develop along the non‐papillary pathway and are at a high risk of progressing towards muscle invasive disease with a substantial risk for the development of distant metastasis 6. The current standard of care for this metastatic disease includes a combination of gemcitabine and cisplatin 7. Apart from this new combinatorial regimen of traditional chemotherapeutic drugs, no new drugs for bladder cancer have entered the market in nearly 30 years. Moreover, a precision medicine paradigm has yet to be investigated for the treatment of this disease. Therefore, there is a clear need for novel agents for intravesical therapy that provide a better treatment after transurethral resection.

An orchestrated control of the cell cycle, including mitotic cell division, is essential, and error‐free chromosomal segregation during cell division is of the utmost importance for the maintenance of correct ploidy and genomic integrity; errors in cell division may result in aneuploidy and cancer 8. For a proper cell division, the daughter cells must receive the correct complement of chromosomes, for which, the following two critical events are required: (*i*) the chromosomes must be equally segregated with the help of the mitotic spindle; and (*ii*) cytokinesis (the process of cell division) must occur without error. Both of these processes are controlled, in part, through the activity of a critical cell cycle regulator, Polo‐like kinase 1 (PLK1), which is a member of a family of conserved serine/threonine protein kinases. The PLK members of the serine/threonine family of kinases play a variety of roles in the G2/M phase transition and have been identified as key regulators of cell mitosis.

Polo‐like kinase 1, the best‐characterized member of the PLK family, is crucial for cell cycle progression and performs multiple functions throughout mitosis 9. Polo‐like kinase 1 overexpression enables cells to override control checkpoints and promotes the transformation of mammalian cells. In line with these observations, numerous studies have revealed that, in normal tissues, PLK1 is found only in proliferating cells, and increased PLK1 gene expression has been described in different neoplasias, which correlates with the prognosis of certain cancers 10, 11, 12. The inhibition of PLK1 induces cell cycle arrest and increases apoptosis in several models. The inhibition of PLK1 also has a cytotoxic effect on cells, and because of this effect, several inhibitors have been developed. Among these inhibitors, the majority of PLK1 inhibitors are ATP competitors that act on the ATP‐binding pocket of the kinase. Recently, Chen *et al*. and Wovkulich *et al*. reported their discovery of a novel class of pyrimidodiazepinones as potent and selective ATP‐competitive PLK1 inhibitors 13, 14. RO3280 exhibits potent antiproliferative activity in a wide range of cancer cell lines, and shows strong anti‐tumour activities in an *in vivo* HT‐29 colorectal xenograft mouse model. However, no study has yet focused on the effects of RO3280 in human bladder cancer cells.

The purpose of this study was to investigate the anti‐cancer effects of RO3280 and study its cellular mechanism in human bladder cancer cells. We observed that RO3280 was highly cytotoxic to bladder cancer cells compared with uroepithelial cells, with IC50 values at single‐digit low nanomolar concentrations. Moreover, our data indicate that RO3280‐mediated PLK1 inhibition resulted in the activation of Wee1, as assessed by the increased Tyr15 phosphorylation of cell division cycle protein 2 (CDC2), unscheduled mitotic entry and apoptosis. RO3280 also induced mitotic catastrophe in bladder cancer cells as demonstrated by the formation of large, multinucleated polyploid cells. Furthermore, RO3280 showed strong anti‐tumour activities in an *in vivo* 5637 bladder cancer xenograft mouse model. Overall, these results suggest that cell apoptosis and mitotic catastrophe account for the anti‐tumour effects of RO3280 as a single agent on bladder cancer cells and represents a promising therapeutic agent in the treatment of bladder cancer.

## Materials and methods

### Cell lines and culture

The human non‐malignant cell line SV‐HUC‐11 and the human bladder cancer lines 5637 and T24 cells were purchased from the Shanghai Institute of Cell Biology, Chinese Academy of Sciences (Shanghai, China) and were cultured in RPMI 1640 (Invitrogen, Grand Island, NY, USA) supplemented with 10% foetal bovine serum (Invitrogen) under an humidified air atmosphere of 5% CO_2_ at 37°C.

### Reagents

RO3280 was purchased from Selleckchem (Houston, TX, USA). Z‐VAD‐FMK was purchased from R&D Systems (Minneapolis, MN, USA). 3‐(4,5‐dimethylthiazol‐2‐yl)‐2,5‐diphenyltetrazolium bromide (MTT) and trypan blue solution were purchased from Sigma‐Aldrich (St. Louis, MO, USA). The Annexin V‐PI Kit was purchased from BD (Franklin Lakes, NJ, USA).

### Protein extraction and Western blot analysis

For protein analysis, tissue samples and cells were lysed in 2% SDS and 0.5‐M Tris‐HCl. Western blots were performed according to standard methods. The following antibodies were used: rabbit polyclonal anti‐MPM‐2 (Abcam, Cambridge, MA, USA); rabbit monoclonal anti‐CDC2 (phospho Y15; Abcam, Cambridge, MA, USA); mouse monoclonal anti‐PLK1 (Abcam, Cambridge, MA, USA); rabbit monoclonal anti‐PARP, rabbit monoclonal anti‐caspase 3 and mouse monoclonal anti‐BubR1 (Abcam,Cambridge, MA, USA); and mouse monoclonal anti‐GAPDH (Sigma‐Aldrich). Signal detection was performed with an ECL system (Pierce,Rockford, IL, USA).

### RO3280 treatment

RO3280 was initially dissolved in dimethylsulfoxide (DMSO) and stored at −80°C and was thawed before use. For all experiments, cells were treated at various concentrations (50, 100 and 200 nM). Corresponding control cultures received an equal volume of solvent. Cells were plated at appropriate densities in culture vessels. Twenty‐four hours after passaging, cells were exposed to increasing doses of 50, 100 and 200 nM RO3280 or DMSO control. At 24 or 48 hrs after treatment, the cells were trypsinized and collected for further analyses.

### 3‐(4,5‐dimethylthazol‐2‐yl)‐2,5‐diphenyltetrazolium bromide (MTT) assay

Approximately 5 × 10^3^ SV‐HUC‐1, T24 and 5637 cells were seeded into 96‐well culture plates. After an overnight incubation, the cells were treated with different concentrations of RO3280. Following incubation for 24 and 48 hrs, cell growth was measured following the addition of 0.5 mg/ml MTT (Sigma‐Aldrich) solution. Approximately 4 hrs later, the medium was replaced with 100 ml of DMSO (Sigma‐Aldrich) and vortexed for 10 min. Absorbance (A) was then recorded at 490 nm by a Microplate Reader 680 (Bio‐Rad, Hercules, CA, USA).

### Cell morphological analysis

Approximately 1 × 10^5^ cells/well cells in 12‐well plates were incubated with or without 50, 100 and 200 nM RO3280, and a equal amount of DMSO was used as a control for 48 hrs at 37°C. At the end of the treatment, cells were examined and imaged under a phase‐contrast microscope at 200× magnification to evaluate morphological changes.

### Colony‐formation assay

After experimental treatment, the cells were trypsinized and reseeded in a 6‐well plate (1 × 10^4^ cells per well) and cultured at 37°C. Colonies were scored 7 days later by staining with crystal violet (Beyotime, Shanghai, China).

### Apoptosis assay using flow cytometry

Apoptosis was assessed using an Annexin V‐coupled fluorescein isothiocyanate (FITC) apoptosis detection kit‐1 (BD Pharmingen, San Diego, CA, USA). Briefly, cells were removed by incubation with trypsin‐EDTA, washed twice in PBS, and resuspended in 1 ml of Annexin V‐binding buffer at 10^6^ cells/ml. Annexin V‐coupled FITC and propidium iodide (PI) were added (each at 5 ml per 10^5^ cells). Samples were mixed gently, incubated for 15 min. at room temperature in the dark, and then subjected to flow cytometry (Becton Dickinson, Oxford, UK) to evaluate the number of apoptotic cells. All experiments were repeated at least three times.

### Cell cycle assay using flow cytometry

The cells were collected and washed with PBS, fixed with 70% cold ethanol at 4°C for 2 hrs, and passed through 70‐μm Falcon Filters (BD Biosciences, Oxford, UK) to obtain a monodispersed cell suspension. The monodispersed cells were incubated with RNase A at 37°C for 30 min. and stained with PI at 4°C for 30 min. (Cell Cycle Detection Kit; BD, USA). Flow cytometric analysis was performed with a FACSCalibur flow cytometer (Becton Dickinson). Finally, the cell cycle distribution was analysed by Cell Quest software (BD, Franklin Lakes, NJ, USA).

### Immunofluorescence

Immunofluorescence analyses were performed with cell lines cultured in 24‐well plates. The primary antibody was a rabbit monoclonal antibody specific for alpha‐tubulin (Abcam). After washing, the cells were incubated with TRITC‐conjugated (mouse anti‐rabbit) secondary antibodies (Beijing Zhong Shan ‐Golden Bridge Biological Technology Company, Beijing, China). The nuclei were stained with DAPI (Beyotime). Immunofluorescence images were viewed with an inverted fluorescence microscope (Olympus, Tokyo, Japan).

### Trypan blue exclusion assay

Later, cells were trypsinized and collected in a 1.5‐ml tube, pelleted by centrifugation and re‐suspended in PBS. A 10‐μl aliquot of cell suspension was removed, and an equivalent 10 μl of 0.4% trypan blue solution was added to the cells. A 10‐μl load of the mixture was placed into the chamber port on the Luna^™^ counting slide. The slide was inserted completely into the slide port of the counter. The cells were counted to determine the total cell growth with the Luna Automatic Cell Counter (Logos Biosystems, Pyungchon Dong Dongan Gu Anyang, Korea).

### Xenograft studies

Briefly, mice (female, 4–5 weeks of age) were purchased from the Beijing Vitalriver Experimental Animal Technical Company (Beijing, China). Cells (5 × 10^6^ cells in 150 μl) were suspended in RPMI 1640 and injected subcutaneously into the flank of each BALB/c nude mouse. On day 5, tumour size was measured, the animals were randomized into two groups (*n* = 15 per group), and RO3280 (40 mg/kg, once every 5 days) treatment was initiated by intraperitoneal injection. The control group was treated with vehicle (1.5% DMSO in PBS). The drug (or vehicle) treatment was performed for 40 days. The length and width of the resulting tumours (in millimetres) were measured every 3 days with callipers. The tumour diameter was measured, and the volume (length × width^2^ × 0.52) was calculated. The mice were humanely killed on day 45, and the tumours were dissected and weighed. Approval to perform the study was obtained from the Medical Laboratory Animal Welfare and Ethics Committee of China Medical University, and the methods were performed in accordance with the approved guidelines. Western blot and immunohistochemistry assays were also performed with these sections. Then, the tumours were fixed, embedded and cut into 3‐μm‐thick sections, which were subsequently stained with haematoxylin and eosin to permit the observation of the tumour margin.

### Immunohistochemistry

Tumours were surgically removed, fixed in a 4% buffered neutral formalin solution and embedded in semisynthetic paraffin. Consecutive 7‐μm‐thick sections were stained with haematoxylin and eosin or haematoxylin alone for assessing microanatomical changes with the Upright Metallurgical Microscope (Olympus). Paraffin‐embedded sections were also stained with mouse anti‐Ki‐67 Ab (1:100, Abcam) following the manufacturer's protocol. After incubation for 30 min. at 25°C with the corresponding secondary biotinylated Ab (goat‐anti mouse IgG, 1:200; Beyotime), the immune reaction was revealed by a DAB kit (Beyotime). Sections exposed to non‐immune sera were used as negative controls. For each tumour sample, Ki‐67‐positive cells were counted in 10 fields of 0.5 mm^2^ from serial consecutive sections.

### Statistical analyses

Statistical analyses were performed with SPSS (Statistical Package for the Social Sciences) 13.0 (SPSS Inc., Chicago, IL, USA). The results are presented as the mean ± S.E.M. unless otherwise stated. A *P* < 0.05 was considered to indicate significant differences. The associations between variables were analysed by Student's *t*‐test and the chi‐squared test. Comparisons were performed for multiple means using an anova after checking for equal variance.

## Results

### Cytotoxicity of RO3280 in human bladder cancer cell lines and a normal uroepithelial cell line

**Figure 1:**
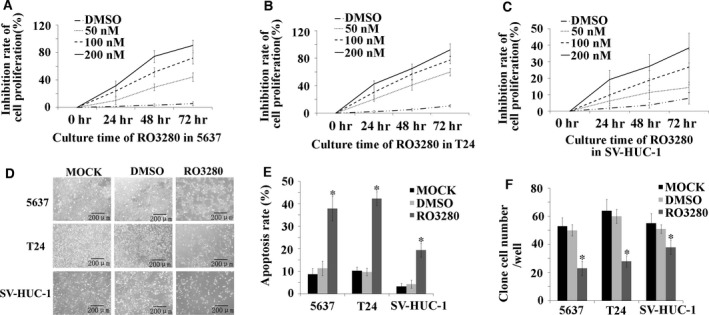
Cytotoxicity of RO3280 in 5637 and T24 cell lines and the SV‐HUC‐1 cell line. (**A**–**C**) The MTT assay was used to examine the proliferation of 5637, T24 and SV‐HUC‐1 cells that were treated with 50, 100 and 200 nM RO3208, treated with an equal amount of DMSO (DMSO) or received no treatment (MOCK). (**D**) Visualization by inverted microscopy (100X) for the morphological effects of RO3280 in 5637, T24 and SV‐HUC‐1 cells. (**E**) Apoptotic effects of RO3280 in 5637, T24 and SV‐HUC‐1 cells were observed using PI staining and are shown using bar graphs (*P < 0.05). (**F**) Bar graphs show the clonogenic survival of 5637, T24 and SV‐HUC‐1 cells exposed to RO3280 (*P < 0.05); the error bars indicate the S.E.M.

The cytotoxic effect of RO3280 was investigated using two human bladder cancer cell lines and the SV40 virus‐transformed uroepithelial cell line SV‐HUC‐1. Using the MTT assay, the results showed that RO3280 had a cytotoxic effect on the 5637 and T24 human bladder cancer cells, with IC50 values of approximately 100 nM (Fig. 1A and B). Moreover, the maximal inhibitory effect was approximately 95%. However, with the same treatment time, the maximal inhibitory effect of RO3280 on SV‐HUC‐1 was only 37% (Fig. 1C). From morphological observations, RO3280 induced more than 70% of 5637 and T24 cells to detach from the dish, but this effect only occurred in approximately 30% of the SV‐HUC‐1 cells (Fig. 1D). This observation suggests that urinary cancer cells are more sensitive to the effects of RO3280 than normal uroepithelium cells. To further identify whether RO3280‐detached cells were alive, the PI staining assay was applied. As shown in Figure 1E, the viability of the cells as assessed using the PI staining assay was higher than that determined with the MTT assay, indicating that some of the RO3280‐detached 5637 and T24 cells were alive. However, according to the results obtained with the PI staining assay, the 5637 and T24 cells were more sensitive to the cytotoxic effects of RO3280 than the SV‐HUC‐1 cells (Fig. 1E). In addition, the clonogenic survival assay was also applied to analyse the cytotoxic effect of transient RO3280 treatment in the two cancer cell lines. Although RO3280 was only present in the medium transiently, at a high dose, RO3280 inhibited the clonogenic survival of 5637 and T24 cells (Fig. 1F).

### Effect of RO3280 on the cell cycle

**Figure 2:**
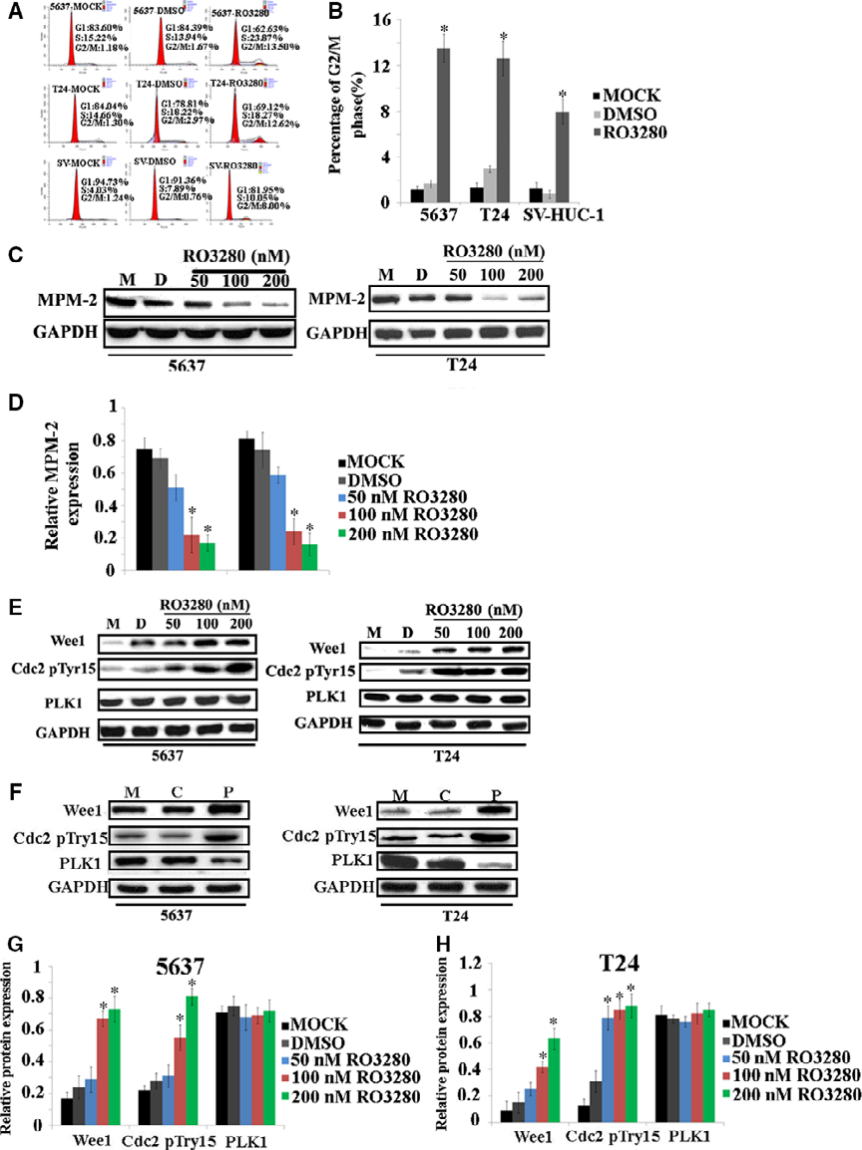
The influence of RO3280 on the cell cycle in 5637, T24 and SV‐HUC‐1 cells. (**A**) Cell cycle distributions of 5637, T24 and SV‐HUC‐1 cells were evaluated using a flow cytometer after RO3280 treatment (100 nM, 48 hrs). (**B**) Histograms represent the cell cycle distribution, and the ratios were calculated (*P < 0.05). (**C**) Band intensities indicate the MPM‐2 protein expression level in 5637 and T24 cells exposed to RO3280. GAPDH was used as a loading control to assure equal amounts of protein in all lanes. (**D**) The ratio of the optical density of MPM‐2 and GAPDH of the same sample by Western blot was calculated and expressed graphically. The data are representative of three individual experiments (*P < 0.05). (**E** and **F**) Band intensities indicate Wee1, the phosphorylation of CDC2 at Tyr15 and PLK1 protein expression in 5637 and T24 cells that were exposed to RO3280 and with treatment of PLK1 siRNA. M: MOCK group, C: Control group, P: PLK1 siRNA group. (**G** and **H**) The ratio of the optical density of Wee1, the phosphorylation of CDC2 at Tyr15, PLK1 and GAPDH of the same sample using Western blotting was calculated and expressed graphically (*P < 0.05); the error bars indicate the S.E.M.

Next, we used a flow cytometer to examine the effect of RO3280 on cell cycle progression. As shown in Figure 2A and B, RO3280 had a dramatic effect on the cell cycle, inducing cell cycle arrest at the G2/M phase in both 5637 and T24 cells. However, we were unsure whether the arrest occurred specifically at the G2 or the M phase. Therefore, using MPM‐2 antibodies, we examined the status of phosphorylated polypeptides, which are found only in mitotic cells. After 48 hrs of RO3280 treatment, at concentrations ranging from 50 to 200 nM, a significant decline in MPM‐2 was detected (Fig. 2C and D). The change in MPM‐2 expression confirmed that the cells were arrested in the M phase and not the G2 phase. In normal human uroepithelium SV‐HUC‐1 cells, RO3280 also induced G2/M arrest, but the proportion of these cells in G2/M phase was lower than that of the 5637 and T24 cells treated with RO3280 (Fig. 2A and B). This result provides further evidence that RO3280 has only a slight cytotoxic effect on SV‐HUC‐1 cells compared with T24 and 5637 cells.

In dividing eukaryotic cells, CDC2 dephosphorylation is essential for mitotic progression 15. Before mitosis, CDC2 activity is suppressed through the Wee1‐mediated inhibitory phosphorylation of Tyr15 and Thr14 residues of CDC2 16. Hence, we investigated whether RO3280 hindered CDC2 dephosphorylation. A shown in Figure 2E–H, with RO3280 treatment, Wee1 expression increased, and there was a high phosphorylation level of CDC2 in 5637 and T24 cells. Thus, the RO3280‐induced G2/M arrest might have occurred through the inactivation of the PLK1/Wee1/CDC2 pathway.

### RO3280 induces the biochemical features of apoptosis in bladder cancer cells

**Figure 3:**
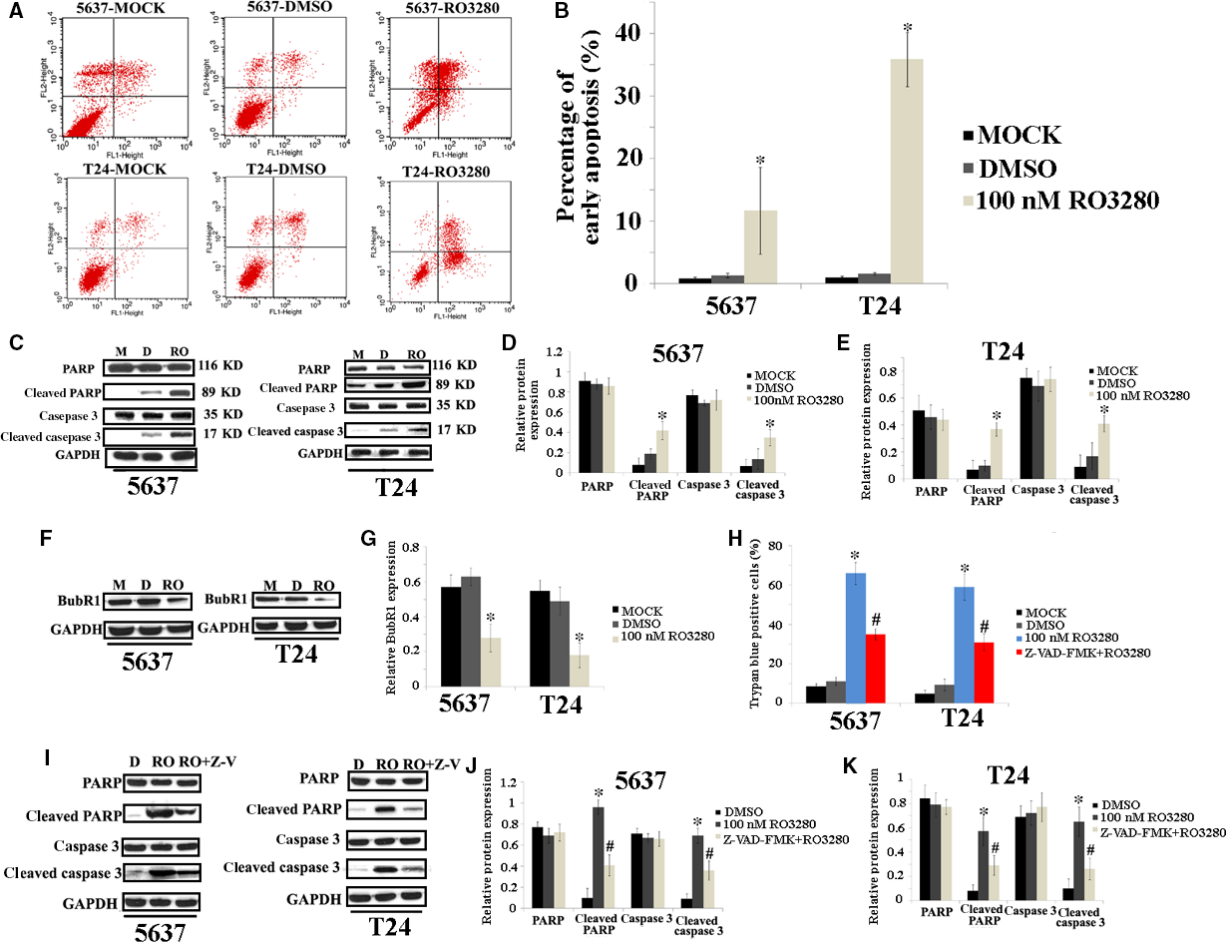
RO3280 induces the biochemical features of apoptosis in bladder cancer cells. (**A**) The ability of RO3280 (100 nM, 48 hrs) to induce apoptosis was quantified using Annexin V‐FITC staining with FACS analysis, where Annexin V^+^ PI ^+^, Annexin V^+^ PI ^−^, Annexin V^−^ PI ^+^ and Annexin V^−^ PI ^−^, respectively, indicate late apoptotic, early apoptotic, necrotic and live cells. (**B**) The bar chart represents the number of apoptotic cells and the ratios were calculated (*P < 0.05). (**C**) Band intensities indicated PARP, cleaved PARP, caspase 3 and cleaved caspase 3 protein expression in 5637 and T24 cells that were exposed to RO3280 (100 nM, 48 hrs). (**D** and **E**) The ratio of the optical density of PARP, cleaved PARP, caspase 3, cleaved caspase 3 and GAPDH of the same sample using Western blotting was calculated and expressed graphically (*P < 0.05). (**F**) Band intensities indicated BubR1 protein expression in 5637 and T24 cells that were exposed to RO3280 (100 nM, 48 hrs). (**G**) The ratio of the optical density of BubR1 and GAPDH for the same sample using Western blotting was calculated and expressed graphically (*P < 0.05). (**H**) 5637 and T24 cells were treated with RO3280 (100 nM, 48 hrs) or RO3280 (100 nM, 48 hrs)+ Z‐VAD‐FMK for 48 hrs, and cell death was determined by trypan blue staining (*, compared with MOCK and DMSO groups; #, compared with RO3280 group; *P* < 0.05). (**I**) Band intensities indicated PARP, cleaved PARP, caspase 3 and cleaved caspase 3 protein expression in 5637 and T24 cells that were exposed to RO3280 (100 nM, 48 hrs) or RO3280 (100 nM, 48 hrs)+ Z‐VAD‐FMK. (**J** and **K**) The ratio of the optical density of PARP, cleaved PARP, caspase 3, cleaved caspase 3 and GAPDH of the same sample using Western blotting was calculated and expressed graphically [*, compared with MOCK and DMSO groups; #, compared with RO3280 (100 nM, 48 hrs) group; *P* < 0.05]; the error bars indicate the S.E.M.

In an additional study, we investigated whether RO3280 could induce apoptosis. The ability of RO3280 to induce apoptosis was quantified using Annexin V staining with FACS analysis. After 100‐nM RO3280 treatment, 5637 and T24 cells showed a higher proportion of early apoptotic cells (10.19% and 36.10%, respectively) compared with the untreated (MOCK; 0.68% and 0.88%, respectively) and vehicle control (DMSO) groups (1.08% and 1.33%, respectively; Fig. 3A and B). Next, using Western blotting, we found that RO3280 induced 5637 and T24 cells to cleave PARP and caspase‐3 (Fig. 3C–E). This observation indicates that RO3280 is effective at inducing apoptosis in 5637 and T24 cells. Therefore, we analysed the M phase check protein BubR1, which is required for M phase arrest and prevents apoptosis 17. BubR1 was highly expressed in 5637 and T24 cells. However, BubR1 was dramatically reduced after 48 hrs of RO3280 treatment in 5637 and T24 cells (Fig. 3F and G). This observation suggests that under RO3280 treatment for 48 hrs, 5637 and T24 cells could exit M phase. To understand the role of apoptosis in RO3280‐treated cells, we further compared the effect of a pan‐caspase inhibitor on RO3280‐induced cell death in these two cell lines. Cells were treated with 40‐nM Z‐VAD‐FMK, and cell death was scored using a trypan blue assay. Z‐VAD‐FMK prevented cell death in 5637 and T24 cells (Fig. 3H). Interestingly, Z‐VAD‐FMK did not completely inhibit cell death, but it did completely block RO3280‐induced PARP and caspase‐3 cleavage in 5637 and T24 cells (Fig. 3I–K), indicating that there are other pathways leading to cell death in addition to RO3280‐induced apoptosis.

### RO3280 induces the formation of multinucleated cells in bladder cancer cell lines

**Figure 4:**
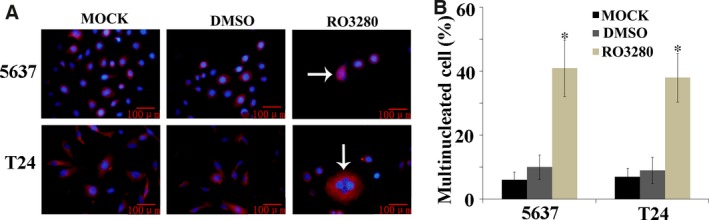
RO3280 promotes the formation of multinucleated cells in bladder cancer cell lines. (**A**) Representative images of cells stained with DAPI (blue) and alpha‐tubulin (red) were examined under a fluorescence microscope (magnification 400×). Enlarged cells containing multiple evenly stained nuclei (multinucleated cells) are characteristic of mitotic catastrophe (white arrow). (**B**) Quantification of the percentage of multinucleated cells based on fluorescence microscopy analysis (the data are presented as the mean ± S.E.M., *P < 0.05).

The formation of giant multinucleated cells is a phenotype of mitotic catastrophe that is induced by ionizing radiation or specific anti‐cancer drugs 18. We investigated whether RO3280 induced giant multinucleation in these two cell lines. As shown in Figure 4A, under the fluorescent field, cells containing the enlarged multinucleated phenotype could be clearly observed after RO3280 treatment. The number of multinucleated cells was calculated by comparing those observed under the fluorescent field to those under the light field. RO3280 at 100 nM, respectively, induced in 41% of 5637 cells and 38% of T24 cells undergo the multinucleated phenotype (Fig. 4B). This observation suggests that 5637 and T24 cells could exit mitosis without cell division. Overall, RO3280 indeed induced a mitotic catastrophe phenomenon in both bladder cancer cell lines.

### RO3280 retards bladder cancer xenograft growth in a nude mouse model

**Figure 5:**
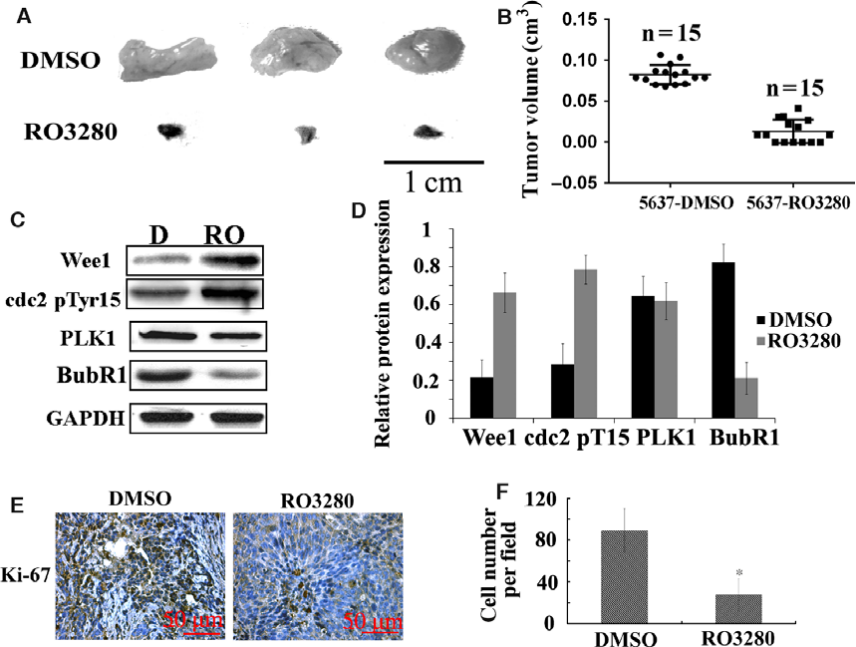
RO3280 retards bladder cancer xenograft growth *in vivo*. (**A**) Representative images of tumours in DMSO‐treated 5637 cell‐transplanted mice (DMSO) and RO3280‐treated 5637 cell‐transplanted mice (RO3280). (**B**) The tumour volumes were measured at the indicated number of days after mice were transplanted with 5637‐DMSO and 5637‐RO3280 cells. The data are presented as the mean ± S.E.M. (**C**) Band intensities indicate Wee1 expression, CDC2 (Thr14/Tyr15) phosphorylation, PLK1 and BubR1 expression in 5637 xenograft with DMSO or RO3280 treatment. The data are presented as the mean ± S.E.M. (**D**) The ratio of the optical density of target protein and GAPDH of the same sample using Western blotting was calculated and expressed graphically (*P < 0.05, the data are presented as the mean ± S.E.M.). (**E**) Cell proliferation was evaluated using Ki‐67 immunohistochemistry in xenografts. (**F**) Statistical analysis of Ki‐67‐positive cells from **C**. The data are presented as the mean ± S.E.M. *P < 0.05.

To assess the anti‐tumour activity of RO3280 *in vivo*, 5637 xenografts were established in BALB/c mice. Thirty mice were randomized into two groups in the 5637 xenograft experiment (15 mice/group). All mice developed bladder cancer xenografts. Treatment with RO3280 (30 mg/kg, once every 5 days) was well tolerated in mice without significant morbidity or mortality. Following 45 days of *in vivo* growth, the xenografts were excised and measured. The volumes of the 5637 xenografts were significantly lower in RO3280‐treated mice compared with DMSO‐treated mice (Fig. 5A and B). Six RO3280‐treated mice had no tumours at 45 days. In the 5637 xenografts, the reduction in the tumour volumes corresponded to an increase in Wee1 expression, CDC2 (Thr14/Tyr15) phosphorylation and the decrease in BubR1 expression (Fig. 5C and D), as determined by Western blotting. The effects of RO3280 on the 5637 tumour biomarker (nuclear for Ki‐67) are presented in Figure 5E and F. These results suggest that RO3280 exhibits pronounced *in vivo* effects against bladder cancer xenografts and is associated with target‐specific pharmacodynamic changes in 5637 xenografts.

## Discussion

Polo‐like kinase 1, a key regulator of cell division in eukaryotic cells, plays critical roles in ensuring a smooth and error‐free progression through mitosis. Polo‐like kinase 1 functions extend past the ‘core’ cell cycle, and the term ‘mitotic kinase’ to describe the activity of PLK1 might not be sufficient 19. Polo‐like kinase 1 expression correlates with a poor prognosis in various cancers 20, 21. Elevated PLK1 levels have been found in many cancers compared with their normal counterpart tissues. In our previous studies, PLK1 expression status closely correlated with important histopathologic characteristics (grades and stages) and the recurrence and metastasis of bladder urothelial carcinomas. Inhibition of PLK1 decreases cell proliferation and invasion by down‐regulating the cancer cell cycle from G1/S to G2/M 11.

Polo‐like kinase 1 is a validated target for cancer, and several PLK1 inhibitors have shown promising results in phase I or II clinical trials. Chen *et al*. and Wovkulich *et al*. reported the discovery of a novel class of pyrimidodiazepinones, which include RO3280, as potent and selective ATP‐competitive PLK1 inhibitors 13, 14. RO3280 exhibits potent antiproliferative activity in a wide range of cancer cell lines. In recent clinical trial studies, the PLK1 inhibitor BI2536 was used in a randomized phase II clinical trial of pancreatic exocrine adenocarcinoma 22. Furthermore, another PLK1 inhibitor, BI6727, maintained broad anti‐tumour activities in several solid tumours 23. However, BI2536 failed to show sufficient efficacy in phase II clinical trials, as there was a low response rate and no significant difference in patient outcome 24. RO3280 is a close analogue to BI2536, but with improved activity against PLK1. Our findings provide a rationale for evaluating the function of this novel PLK1 inhibitor in bladder carcinoma. RO3280 holds promise as a novel molecularly targeted treatment drug for bladder carcinoma. Therefore, the objective of this study was to determine the pre‐clinical effects of targeting PLK1 in bladder cancer *in vitro* and *in vivo*.

RO3280 showed cytotoxicity towards two bladder cancer cell lines at low concentrations. In this study, the effectiveness of RO3280 in suppressing bladder cancer cell growth, while sparing the inhibition of the non‐tumorigenic SV‐HUC‐1 cell line, shows its potential to serve as a chemotherapy drug. The two bladder carcinoma cell lines displayed low EC50 values and expressed high levels of PLK1, the molecular target for RO3280. With regard to SV‐HUC‐1, which only expresses low levels of PLK1, RO3280 had little effect on its growth. In contrast, PLK1 is expressed in highly proliferative cancer cells and is regarded as a cell proliferation marker 25, 26. These findings further support the potential efficacy of using RO3280 for the treatment of bladder carcinoma. The RO3280 compound did not induce any noticeable acute toxicity in mice. However, due to inherent limitations in the use of mouse models, the primary hematologic toxicity observed in patients treated with other PLK1 inhibitors 27 could not be evaluated, but remains a potential side‐effect that warrants further scrutiny in the future.

Polo‐like kinase 1 also plays an important role in maintaining genomic stability during DNA replication and the DNA damage checkpoint. Inhibition of mitosis can result in multinucleated cells. The multinucleated cells ultimately undergo apoptosis 28. Therefore, the G2/M checkpoint is a good target for treating cancer. In our experiments, RO3280 inhibits PLK1 kinase activity in cancer cells and induces G2/M arrest in bladder carcinoma cell lines. This finding could explain the low toxicity of RO3280 in mice, as the normal cells are spared.

To investigate the mechanism through which mitotic arrest occurred, we assessed the expression of important controllers of the cell cycle at the G2/M transition. Polo‐like kinase 1 is a key regulator modulating different signalling pathways to control the G2/M transition. Polo‐like kinase 1 induces Wee1 phosphorylation and thus facilitates its degradation through proteasome‐dependent degradation after ubiquitination by the E3 ubiquitin ligase 29. Wee1 is a tyrosine kinase with a key role as an inhibitory regulator of the G2/M checkpoint that precedes entry into mitosis. Wee1 can phosphorylate Cdk1 and inhibit its function to drive cell cycle progression 30. Early studies showed that cyclin B is up‐regulated in PLK1‐knockdown nasopharyngeal carcinoma cell lines 31. However, in this study, RO3280 increased the expression level of Wee1 and led to the high phosphorylation of the Wee1 substrate CDC2. The cyclin B protein level was decreased in the RO3280‐treated cells, which may be related to cell apoptosis 32.

Loss of the G2/M checkpoint prematurely transitions cells that are genomically unstable into the M phase, leading to widespread cell death 33. The molecular mechanisms through which RO3280 causes cell death are complex and are most likely mediated through caspase‐dependent and ‐independent pathways 14. In this study, we showed that RO3280, respectively, induced 59% and 65% of the 5637 and T24 cells to apoptosis. Furthermore, RO3280 could make PARP cleavage, caspase‐3 activation and BubR1 degradation. Furthermore, RO3280‐induced cell death could be partially reversed by a pan‐caspase inhibitor in both of these cell lines, indicating that RO3280‐induced cell death is partially mediated by the caspase pathway. RO3280 also induced the formation of giant multinucleated cells in 5637 and T24 cells. These multinucleated cells, and the abnormal separation of chromosomes, will lead to mitotic catastrophe, and this effect may be used in anti‐cancer therapy 34. Currently, it remains unclear whether mitotic catastrophe activates non‐caspase genome digestion, but non‐caspase‐induced genome digestion has been reported 35. Our data reveal that non‐caspase‐induced cell death is an important mechanism in RO3280‐induced cell death.

Here, we used the murine xenograft bladder tumour model to analyse the *in vivo* effects of intravesical RO3280 therapy. In addition to its ability to hinder human bladder tumour cell growth *in vitro*, RO3280 has similar activity against human bladder xenograft tumours growing *in vivo*. Here, we demonstrated that this anti‐tumour efficacy extends to bladder tumour xenografts growing in nude mice that were treated with RO3280. RO3280 significantly extended the survival of the mice compared with the untreated control mice. In the 5637 xenograft model, the reduction of the tumour weights corresponded to the increase in Wee1 expression, CDC2 (Thr14/Tyr15) phosphorylation and the reduction in PLK1 expression. In another experiment, RO3280 displayed robust anti‐tumour activity that ranged from tumour growth inhibition when dosed once every 5 days at 40 mg/kg to complete tumour regression when dosed more frequently.

In summary, our findings provide a mechanistic insight into the effects of RO3280 in two human bladder cancer cell lines and a therapeutic evaluation of its application *in vivo*. RO3280 suppressed bladder carcinoma growth by inducing G2/M arrest and mitotic arrest and ultimately induced apoptosis and mitotic catastrophe through inhibiting PLK1 activity, which activates the signalling molecules that are negatively regulated by PLK1. Our data support further study of RO3280 as a potential agent for chemotherapy against bladder cancer.

## Conflict of interest

None.
